# Reasons and Strategies for Privacy Features in Tracking and Tracing Systems—A Systematic Literature Review

**DOI:** 10.3390/s21134501

**Published:** 2021-06-30

**Authors:** Christian Jandl, Markus Wagner, Thomas Moser, Sebastian Schlund

**Affiliations:** 1 Department Medien und Digitale Technologien, University of Applied Sciences, 3100 Sankt Pölten, Austria; markus.wagner@fhstp.ac.at (M.W.); tmoser@fhstp.ac.at (T.M.); 2 Institute of Management Sciences, University of Technology, 1040 Vienna, Austria; sebastian.schlund@tuwien.ac.at

**Keywords:** asset tracking, employee monitoring, localization systems, privacy by design

## Abstract

In the course of the digitization of production facilities, tracking and tracing of assets in the supply chain is becoming increasingly relevant for the manufacturing industry. The collection and use of real-time position data of logistics, tools and load carriers are already standard procedure in entire branches of the industry today. In addition to asset tracking, the technologies used also offer new possibilities for collecting and evaluating position and biometric data of employees. Thus, these technologies can be used for monitoring performance or for tracking worker behaviour, which can lead to additional burdens and stress for employees. In this context, the collection and evaluation of employee data can influence the workplace of the affected employee in the company to his or her disadvantage. The approach of Privacy by Design can help to benefit from all the advantages of these systems, while ensuring that the impact on employee privacy is kept to a minimum. Currently, there is no survey available that reviews tracking and tracing systems supporting this important and emerging field. This work provides a systematic overview from the perspective of the impact on employee privacy. Additionally, this paper identifies and evaluates the techniques used with regard to employee privacy in industrial tracking and tracing systems. This helps to reveal new privacy preserving techniques that are currently underrepresented, therefore enabling new research opportunities in the industrial community.

## 1. Introduction

The tracking and tracing of objects has found widespread use in transport and for logistic purposes over the past twenty years. Detailed information about the position and status of an object allows for better planning and scheduling, thus enabling leaner and more flexible processes, stock reductions and even new business models. For example, real-time load tracking services have been successfully implemented and provide a clear benefit for logistics companies [1]. Obviously, the concept of tracking and using this information headstart to optimize processes and to create services is of interest for various fields apart from logistics. With the price decline of geographical tags (geotags), sensor integration and convenient and accessible mobile networks almost everywhere, the investment costs have fallen to a very affordable range. In addition to outdoor solutions, indoor positioning systems are rapidly gaining importance in the industry. Back in 2016, 10 percent of German manufacturing companies already had respective solutions in place [2]. With the rise of tracking and tracing systems, issues other than the economic benefit have to be taken into account. One of the issues that arise within the industrial use context is the exposure of personal data to a technical system via a (potentially insecure) communication channel.

These personal data might be direct, such as information about a person’s position and conditions, tracked for purposes of safety or convenience.

A second privacy-related context is the indirect tracking of personal data via process-related context. In the initial example of load tracking, a moving load allows the tracking of the driver and, e.g., his or her (productive) working hours in an indirect but very straightforward manner.

In our private lives, these issues are far from new as many location-based services offer their benefits by combining an object’s or person’s geotag with information that enhances this context. The difference here is that the decision to expose personal data within an employer-employee relationship is far from voluntary and is expected either directly (by oral agreement) or indirectly (by a signed work contract). Thus, compliance with the use of personal data for tracking purposes always refers to the business context. In Germany, Austria and large parts of Western Europe, the use of personal data of employees to optimize the employer’s processes is a strongly regulated area due to the fact that within these societies, privacy issues are considered to be of great significance and employee representatives therefore are very sensitive regarding the topic. The implementation of asset tracking and tracing systems (ATTS) precisely enables one of the most critical issues within this context: the tracking of performance data of individual employees. Because of this situation, the involvement of employee representatives and the proper design of the system use and limitations of data use have to be considered prior to an implementation of asset ATTS within the industry. Therefore, Privacy by Design (PbD) could be considered a key enabler to address this open issue and to unleash the full potential of asset tracking technologies within industrial contexts.

In terms of key contributions, this work provides a detailed survey of the most relevant literature about ATTS for employee and object tracking that have been proposed in the literature between 2014 and 2021.

1. Evaluating existing ATTS using existing evaluation guidelines [3] to highlight their pros and cons concerning the privacy features of the systems.
2. Discussing different guidelines and standards that can be used for indoor localization systems in detail with a special focus on privacy.
3. Providing the pros and cons of different frameworks and highlighting their suitability and challenges for employee tracking.
4. Presenting challenges and opportunities for future research and discussions at the end of the paper.

The remainder of this paper is structured as follows: after discussing relevance and background in Section 2, the research method and the process of finding and selecting suitable articles are described in Section 3. Section 4 and Section 5 present and compare the surveyed approaches and describe the criteria used for comparison. The paper concludes in Section 6 with a discussion of the findings and a presentation of future research challenges in the field of privacy preserving ATTS.

## 2. Relevance & Background

To open up this interdisciplinary field of research, it is important to gain knowledge of the entire range of ATTS and possible privacy issues. On the one hand, it is important to recognize the problems of technologies used in ATTS with respect to privacy, since these often lead to unexpected privacy issues. Therefore, Section 2.1 deals with the basic properties of Wireless Sensor Networks (WSN) that serve this purpose, and localization techniques in Indoor Positioning Systems (IPS) are presented. On the other hand, it is important to have a good understanding of privacy in order to understand the problems of employee surveillance through ATTS. Therefore, in Section 2.2 different approaches related to privacy are discussed and the privacy of employees in particular is examined.

### 2.1. Indoor Positioning Systems and Asset Tracking

In general, Tracking and Tracing refers to the method of tracking physical items by scanning bar code labels or tags in WSN. These tags are attached to the items of interest and transmit their location to a receiver. In outdoor environments the Global Positioning System (GPS)—a Global Navigation Satellite System (GNSS)—is mostly used for Tracing [4]. However, since no GPS signals can be received in indoor environments, it is necessary to set up Indoor Positioning Systems. IPS have become very popular in recent years for solving real-world problems such as: indoor navigation; location detection of products stored in a warehouse; location detection of medical personnel or equipment; or firemen location detection in a burning building [5].

A wireless sensor network (WSN) is an infrastructural environment based on sensor nodes in an infrastructure-based or in a self-organizing ad-hoc network for sensor data collection and transmission. It is deployed in the monitoring area by a large number of low-cost micro sensor nodes, through wireless communication to form a multi-hop self-organized network system. Its purpose is to collaborate perception, acquisition, and processing of network covered area of information of perceived objects, and send these data to a centralized server. In particular, when collecting data from moving objects, this technology has several advantages such as: (1) no wiring between required for tracking; (2) Self-organized infrastructure; and (3) low operational maintenance [6].

For the required WSNs for indoor Tracking and Tracing, common radio technologies such as Ultra Wideband (UWB), Bluetooth (BT) [7,8,9], WiFi [10], ZigBee [11] and Radio Frequency Identification (RFID) [12] are used.

To track or trace an object (person or asset), it must be equipped with a sensor-tag that is able to communicate with the WSN. To estimate a location of a tracked object various signal metrics, such as for example Received Signal Strength Indicator (RSSI), Time of Flight (ToF),Finger Printing and Angle of Arrival (AoA), are used [13]. However, the indoor location is still a challenge for different wireless signals. In Table 1 an overview of the most common technologies in relation to the location precision, drawback and privacy risks according to Zafari [13] are presented.

#### Asset Tracking and Tracing Systems (ATTS)

The term ATTS is used in this paper to provide a common term for the tracking and tracing systems evaluated in this literature review. ATTS are used to track, store, process and provide the position data of objects. Therefore position data from the real world is transferred to the digital world without interaction in the sense of real world awareness for the purpose of continuous traceability near realtime [20].

**Figure 1:**
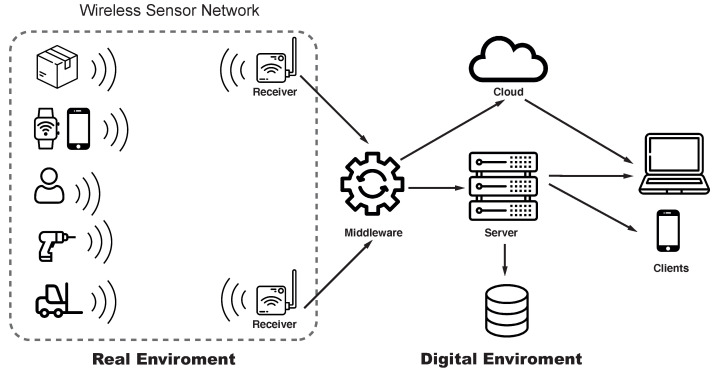
Exemplary illustration of an ATTS architecture.

As shown in Figure 1, an ATTS basically consists of two main components: a WSN, which is responsible for the communication between the tracked objects by means of tags and readers, and an IT system, which consists of middleware, server and client. Additionally, this system may include a cloud or database component for storing or processing the position-data.

Systems currently in the market send the received data of the monitored object to a back-end server without checking whether the movement of the object is relevant in relation to the production environment. Data from employees’ mobile devices, e.g., devices such as smartwatches or fitness wristbands, are also being transmitted and processed. This leads to an increased volume of data within the network and makes it more difficult to evaluate the obtained data [21].

### 2.2. Privacy by Design in the Working Environment

Privacy by Design (PbD) is a term describing a fundamental basic principle in constructing software and systems: privacy is not an add-on but a fundamental architectural principle. It was first used by Langheinrich [22] and is based on ideas that date back to the 1960s, in particular ideas about fair information practices from the US Data Protection Act of 1974, which were later codified in the OECD Guidelines [23]. This directive was implemented in an extended version in the European Union Directive 95/46/EC on the protection of individuals with regard to the processing of personal data and on the free movement of such data [23]. The framework from Langheinrich [22] was later refined into seven foundational principles by Cavoukian et al. [24]. These still rather abstract design principles were refined in subsequent years in order to provide more practical guidelines for actually delivering on the promises of PbD.

While a lot of work has already been done in the field of the Internet of Things (IoT) and User Privacy, the privacy of employees in interacting with IoT systems in the working environment has received less attention. Thus, this paper aims to close this gap by presenting current trends and developments in this important area.

In recent years, it has become easier for employers to obtain information about their employees. Digitization and automation have made employee data highly exposed, e.g., by tracking performance indicators, the number of breaks they take, and in which ways they cooperate with others. Furthermore, many employees are not aware of the fact that human resources departments also regularly use social media to find out more about their employees. This reduces anonymity at the workplace and employees must expect to be evaluated using these measures [25].

Additionally, the shift towards flexible working times and non-permanent employment relationships puts employees in an increasingly unfavourable position when it comes to protecting their personal data. This development is supported by increased teleworking and the simultaneous use of mobile phones and laptops provided by the company. Employees can no longer be sure which data will be forwarded to the employer. For example, GPS monitoring on the smartphone could constantly transmit the employee’s location and violate the employee’s location privacy. By receiving tools for working from home, the employees also find themselves in a situation where the boundaries between private life and work are becoming increasingly blurred [25].

Furthermore, the use of ATTS with all their advantages is an additional intrusion into the privacy of employees. ATTS also offer new possibilities for recording and evaluating the temporal position of employees in the company. On the one hand, this can be done consciously (actively) in order to increase occupational safety at hazardous workplaces, such as in mines or chemical factories, or especially in the health sector, to localize employees or patients in the case this is required [26,27]. These ATTS usually rely on common radio standards. It is also possible to obtain (passive) position data from employees’ mobile devices (mobile phone, smartwatch, fitness wristband) without their consent [28]. Data on the location of users is very sensitive information, as it encroaches on the privacy of users. Companies that evaluate these data could, for example, determine how much time an employee spends at his or her workplace and use this information as an argument for dismissal. To protect the privacy of employees in the company, it is necessary to consider the PbD approach during the development and operation of ATTS.

### 2.3. Related Work

Even though privacy preserving tracking systems are a prevailing challenge and a worthwhile application domain for Industrie 4.0 and healthcare, we could not identify any academic work surveying this field from a privacy preserving perspective. In the related area of IoT applications, privacy is surveyed by Aleisa and Renaud [29], who describe 122 different IoT applications divided into 5 different groups of privacy protection themes such as cryptography techniques, data minimization, access-control, privacy awareness and others. This review was very helpful to identify the different protection themes and which protection themes are useful for Iot application in general. Ref. [30] did a survey on security and privacy issues in IoT. Overall they addressed privacy concerns and mapped them into four clusters, such as privacy in device, privacy during communication, privacy in storage and privacy at processing. These clusters are not very suitable to transfer it to the context of tracking people or objects, because therefore only the clusters of privacy of storing and processing can be measured. Ref. [31] surveyed publications aimed at improving security and privacy in implantable medical devices and health-related body area networks, providing clear definitions and a comprehensive overview of the problem space. They identified three broad research categories aimed at ensuring the security and privacy of the telemetry interface, software, and sensor interface layers. For body implementations, as well as for tracking humans, it is important that the highest possible privacy level is considered standard.The classification of the privacy threads in implantable medical devices and body area networks helped to classify the privacy issues in Section 4, where we clustered typical reasons for privacy features in ATTS. Ref. [32] provides a compact overview of the applications of privacy-preserving techniques to supply chain collaboration among multiple parties. That literature reviews perceives the privacy problems from the process level. This work was helpful to obtain insights into the provision of information in the different roles in the supply chain. Tracking systems are often part of tools that help to manage the supply chain, therefore it was very interesting to investigate their approach. In [33], Loukil et al. report a systematic literature review of privacy preserving solutions used in Cooperative Information Systems in the IoT field. They distinguished between domains (personal and home, government and industry) and in four types of architecture for IoT applications. They focused on which privacy preserving techniques are mostly used in published papers. The research focus is quite similar to this review. However, we endorse a bottom-up approach of privacy concepts for tracking systems. Ref. [34] surveyed security and privacy issues in IoT. To respect privacy, the following levels (1) data collection, (2) data aggregation and (3) data mining must be considered. The have a very architectural approach, where they identify different security and privacy problems of IoT. This review also provides categories of privacy issues we use in that work.

## 3. Research Method

To assess the limits of privacy that are potentially violated by the use of tracking systems, a systematic literature review [35] was conducted to be comparable to a narrative style. It is able to identify the areas covered by existing research or to reveal the gaps as well. Furthermore, it approaches the existing literature from different perspectives and facilitates the acquisition of new knowledge. The following research questions are investigated in this study:

**RQ1:**  What are the most common reasons to implement privacy features in ATTS?
**RQ2:**  Which approaches exist to evaluate the privacy of users in ATTS?
**RQ3:**  What are the most frequently used strategies implementing privacy features in ATTS?

**Figure 2:**
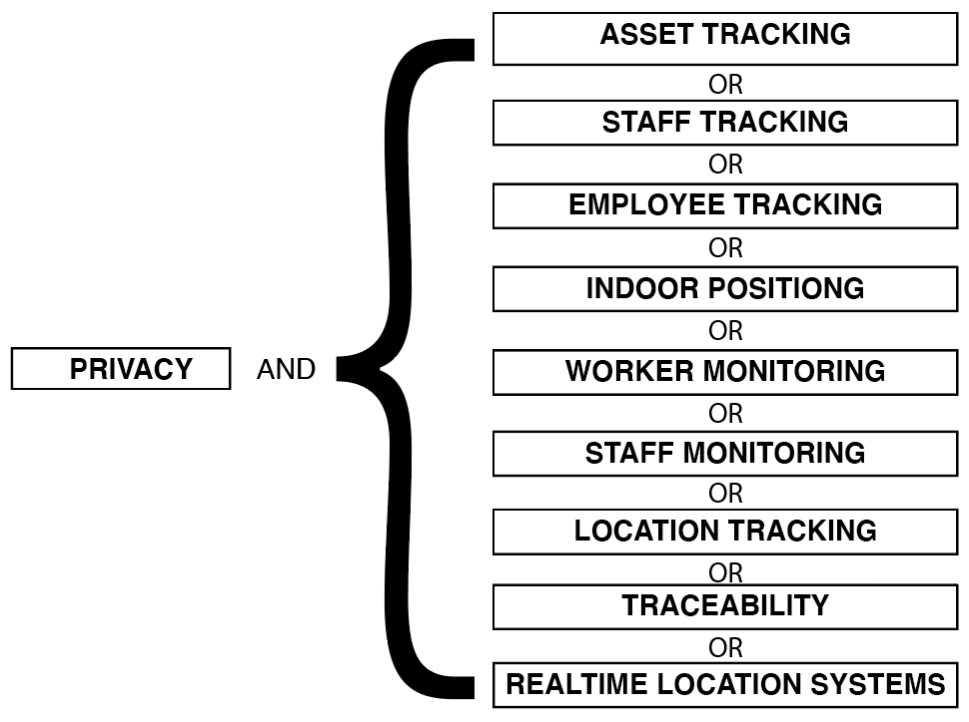
Graphical presentation of the used search term.

To obtain a comprehensive overview of privacy preserving methods supporting tracking systems for industrial and healthcare environments, the following digital libraries were searched: IEEE Xplore (https://ieeexplore.ieee.org/Xplore/home.jsp (accessed 25 April 2021)), ACM digital library (https://dl.acm.org/(accessed on 12 April 2021)), Springer Link (https://link.springer.com/(accessed on 05 April 2021)) and Science Direct (https://www.sciencedirect.com/ (accessed on 31 March 2021)). All searches were based on title, keywords and abstract. The search took take place between October 2019 and May 2021. For all sources, the following search strings were used: *privacy* AND *asset tracking* OR *staff tracking* OR *employee tracking* OR *indoor positioning* OR *worker monitoring* OR *staff monitoring* OR *location tracking* OR *real time location systems* OR *tractability*. Figure 2 shows an graphically presentation of the used search term.

This search resulted in 900 articles, to which we applied the following criteria. We looked for ATTS which implemented privacy features and had a strong emphasis on this. A lot of the papers we found dealt with the topic in a wider context such as general guidelines, specific privacy issues without ATTS or privacy preserving protocols. Furthermore, many papers on tracking systems were found in which the search terms were present but the topic of privacy was not discussed in detail. This explains the actually very high number of excluded papers. In addition, many systems were found that use GPS as a technology, and since this work only deals with indoor ATTS, these sources were also excluded. More detailed information about the search process can be found at the repository of that article (https://github.com/CJ1979/sensitrack (accessed on 30 April 2021)). Generally, peer-reviewed articles dealing with the mentioned topics published between January 2014 and May 2021 in international conferences and journals were considered. In addition it was searched in the proceedings of important privacy and security conferences, such as the Network and Distributed System Security Symposium (NDSS) and USENIX Security Symposium. A careful selection of the most relevant literature was carried out. To ensure that all relevant papers were found, the references of the first 15 peer reviewed found journal articles were scanned to find further possible publications. We also checked conferences and journals where these publications were listed to find additional material. Furthermore, relevant patents were checked for resulting publications. All these measures did resulted in three new articles. Thus, We identified 18 papers and articles that matched the mentioned criteria. In Table 2 an overview to the inclusion and exclusion criteria can be found.

The data analysis was divided into two parts: (1) the reasons why privacy issues were concerned in the ATTS, and (2) which privacy features were implemented.

The selected articles were carefully read, categorized, and analyzed according to the evaluation guidelines of Perera et al. [3] to answer the RQ3. This guidelines were chosen because it is based on already established principles and strategies such as Hopeman [36] and Cavoukian [24] but allows a more detailed analysis of the data- and process-related privacy considerations of an IoT system. The following codes are used: If a guideline is not supported at all or requires substantial effort to support a certain functionality, it is marked as NO-SUPPORT (**✗**). When a particular guideline is not applicable for a given system, it is marked as NOT-APPLICABLE (−). When a system did not support a guideline but provides a mechanism to support user privacy protection through extensions, it is marked as EXTENDABLE (**✓**). If a particular guideline is fully supported in taking steps to protect user privacy as explained in the principles by a given system, it is marked as FULLY-SUPPORTED (**✓✓**). This was done for every guideline, grouped on the strategies by Hoepman [36], for gaining a better overview of the different guidelines. It should be mentioned that Perera et al. [3] divided the data life cycle into five phases. Within each node, data move through five data life cycle phases: Consent and Data Acquisition, Data Pre-Processing, Data Processing and Analysis, Data Storage, and Data Dissemination. In this work, we skipped this step to provide a better overview and comparability of the evaluated systems.Table 3 shows all articles that were chosen for the analysis.

## 4. Reason and Need for Privacy Features in Tracking Systems

Even though PbD is an integral part of data protection legislation in Europe, this concept is not used in many projects yet. Therefore, it would be important, especially in scientific work, to emphasise the threat to privacy in prototyping more strongly. Particularly in sensitive areas such as ATTS, privacy should be taken into account as well. As already mentioned in Section 3, a lot of research is done to address the challenges including energy efficiency, accuracy or security but the important topic of privacy in combination with ATTS is currently not widely researched. Reasons for this could be a lack of awareness or the fact that most of the guidelines are abstract and it takes time to internalize the strategies. This research project examines the stated reasons as to why privacy has been implemented in order to help future development teams identify areas of application where it is particularly important to consider user privacy. Therefore, we collected all arguments from the authors as to why privacy features were implemented in those ATTS and generated the following categories (see Table 4): (1) *Information Leakage or Modification* (2) *Law Compliance* (3) *Sensitive Data Processing* (4) *Data Forwarding* (5) *Data Misuse* (6) *Non Privacy-Compliant Commercial Systems*. In Table 4 a overview between the publications an the respective reasons for privacy features can be seen.

**(1) Information Leakage or Modification:** Information Leakage or Modification refers to the fact that it should not be possible to leak or modify personal data collected by ATTS or information that is stored on tracked tags. For example, the cloning or eavesdropping of tags leads to information leakage, which reveals some information related to the object or the owner [12,51,52]. To build trust in ATTS, it needs to be ensured that tags can be read and rewritten only by authentic readers. These is important especially for RFID applications where memory size is limited and the cloning or rewriting of tags is a possible attack scenario [47].

**(2) Law Compliance:** An important reason privacy is addressed in ATTS is to reach legal certainty. The General Data Protection Regulation (GDPR) is a European Union regulation that harmonizes the rules for the processing of personal data by data processors throughout the European Union. The intention is to ensure the protection of personal data within the European Union. Companies are urged to process the personal data of customers and employees in such a way that no violation of privacy can occur. Non-compliance can result in heavy penalties. This means that if companies or institutions use ATTS, they need to make sure that the applied tracking system has no negative impact of the privacy of the users.

**(3) Sensitive Data Processing:** Especially when ATTS are used to record movement data of persons or metadata could be used to trace back to persons, it is necessary to take special precautions to protect the privacy of these persons. Particularly sensitive applications are, e.g., the use of tracking systems in the health sector where patients and staff are tracked and the recorded patients’ data are linked to their health records, or the industrial environment where the collected data can also be used for analysis in business decisions [41]. In addition, the collected data also enable the electronic monitoring of the performance and behavior of employees. This leads to a subjective feeling of supervision among employees and creates increased uncertainty [53].

**(4) Data Forwarding:** In some cases, it may be necessary to work with third parties, e.g., in supply chain management or the healthcare sector. This requires secure interfaces to ensure the privacy-compliant exchange of data. RFID tags, barcodes and QR codes are not suitable for securing a physical asset in a tracking process because they can be copied. Looking at food supply chains, this requirement includes, for example, ensuring that the cold chain has been adhered to for perishable products, as well as tracing the food back to its farm origins. A challenge for tracking products is the individual feature to be recorded. The major drawback of using RFIDs is that they can be copied, so it would be possible to create a fake history of a product. The idea here is to have a trustworthy middleware that all participants can trust [46].

**(5) Non Privacy-Compliant Commercial Systems:** Commercial systems are usually developed for a wide range of application areas. The reason is that suppliers want to provide a wide range of use cases and the development for special applications would be very cost-intensive. Therefore, commercial vendors tend to focus on costs, scalability, accuracy and technology standards. The vendors expect the system operators to use the measures in order to protect privacy. In many cases, however, this is not possible due to the systems’ architecture. In other words, it is not possible to react to specific privacy requirements in an ATTS. One problem, for example, could be that BT-based systems detect all peripherals in the environment, so the smartphones and fitness wristbands of the employees are also tracked unintentionally [7].

## 5. Privacy Strategies

This section explains various principles and guidelines from literature based on the privacy strategies of Hoepmans et al. [36], which are also used by the European Union Agency for Network and Information Security (https://www.enisa.europa.eu/publications/privacy-and-data-protection-by-design (accessed on 31 March 2021)) for their PbD policy in combination with other guidelines and principles. Afterwards, we take a closer look at which ATTS from the literature search have implemented those principles. Basically, there are two types of strategies: (1) *data-oriented* and (2) *process-oriented* strategies. Both types are needed to provide full privacy protection.

### 5.1. Data-Oriented Strategies

The following four data-oriented strategies can support unlinkability and primarily address the principles of necessity and data minimization.

#### 5.1.1. Strategy 1—MINIMIZE

The first data-oriented strategy is the most basic but valuable for preserving privacy. It states that the data which are processed should be restricted to the minimum possible amount. This means that attempts are made to collect as little personal data as possible to achieve the required result [36]. The PbD framework for IoT applications introduced by Perera et al. [3] also includes the following sub-guidelines to better describe this strategy: (A) minimize data acquisition of data types, duration and sampling frequency, (B) minimize the number of data sources in IoT applications to reduce the possibility of privacy violations by third parties, (C) minimize raw data intake and consider converting raw data into secondary context data, (D) minimize knowledge discovery—which suggests that only the personal data that are necessary to achieve the primary objectives should be collected, (E) minimize data storage, and (F) minimize data retention periods, which suggests to keep the duration of data storage as low as possible. Long retention periods provide more time for third parties to attempt unauthorized access to data [3].

Furthermore, the ISO 29100 standard lists the principles of collection limitation and data minimization, which are based on the “minimize data" strategy. Collection limitation means to limit the collection of personal identifiable information (PII) within the bounds of applicable law and strictly necessary for the specified purpose. The data minimization principle of the ISO 29100 goes one step further and strictly minimizes the processing of PII [54]. This can be done through limit the stakeholders, who have access, make sure that one only grant access if it is necessary for the conduct of his/her official duties and delete the data whenever the processing of PII has expired. Colesky et al. [55] defined that these two principles of the ISO 29100 should be combined in the MINIMIZE strategy to avoid a further strategy limitation deviating from the ISO standard.

As can be seen in Table 5, an attempt to implement the MINIMIZE strategy was made in nearly all of the examined papers. This is also obvious as this strategy is the most important and the one that can be implemented most easily. To implement this strategy in the design of an ATTS, it should be defined at an early stage which goals should be achieved through its application. In the second step, it is necessary to decide which data are required to solve the task, for how long they should be stored, and which sampling frequency is required. In [45], for example, the position of the tracked object is only stored in the database for 24 h, while older location data are deleted. Ref. [37] specifies that only the general zone is saved in the database, not the exact coordinates. In the work of [38], there is no link between the name of the person and the ID of the tag. Only the type of employment is stored on the tag.

In this way, it can be avoided that individual persons are tracked. Ref. [10] presents a system that tracks patients in the hospital. To protect their privacy, all determined position data are deleted when the patient is discharged from hospital. Then the process of solving the intended objective can start. Jandl [7,41] implemented this by whitelisting tags directly at the receiver node. Data from all other Bluetooth devices such as smartwatches and smartphones of the employees, which should not be tracked, were deleted directly at the receiver node and thus not forwarded.

#### 5.1.2. Strategy 2—HIDE

Basically, this strategy describes that the displaying of all personal data and their interrelationships should be avoided. This is often overlooked but very important [36]. For example, if it is possible to link RFID tags or network identifiers such as MAC addresses to an identifiable person, it is possible to track that person throughout the entire tracked area. The core function of the HIDE strategy is to achieve unlinkability and unobservability [56] of a person’s data. For Perera et al., the terms encryption, anonymization and hidden data routing are especially important in this context. Encryption should be used in every possible process step such as communication, processing and storage of data. Currently it is the state of the art to use encrypted communication such as symmetric encryption, e.g., AES256 in the application layer or Secure Sockets Layer (SSL) in network communication.Thus the HIDE strategy also includes access control and data sharing. In ISO 29100, the principle Information Security covers this topic, and adhering to this principle means limiting access to PII to individuals who require access in order to perform their duties [54]. Coleski et al. extended the strategy to: “Preventing exposure as much as possible by mixing, obfuscating, dissociating, or restricting access to any storage, sharing or operation on personal data, within the constraints of the agreed upon purposes” [55].

On the other hand, anonymization has to be ensured as early as possible in the data life cycle. PII should be removed before the data are processed or forwarded to another processing step. Hidden data routing means to use tools that hide the real network address or exchange it with self-created and changing IDs. Ref. [12] implemented unlinkability by changing the RFID tag’s anonymous identity with every new reader interrogation, so it becomes impossible for the attacker to trace the tag by its ID query. Ref. [40] argues that their system does not compromise user privacy because it does not require user authentication. However, it should be mentioned that MAC addresses of peripheral devices can also be traced, which is why conclusions about the behavior of the users are possible [17].

Ref. [49] use k-anonymity [57] to preserve privacy against client-side and server-side attacks by guaranteeing that the tracked person’s location is guessed with a maximum probability of k${}^{-1}$. To face the unique requirements in crowd sensing systems, e.g., user privacy and data utility [44] proposes a concept of mix zones to provide trajectory privacy while achieving high spatial accuracy and only operates over encrypted locations such that traces of the participants are never shared in clear with anyone.

To provide healthcare data via RFID in a privacy-preserving way, ref. [58] propose the concept of tag anonymity. This means that attackers cannot define the identity of the tag even if they illegally access the relevant information. The secret of the tag is never disclosed publicly because it is never communicated directly between the tag and the reader, and it is only stored locally in the tag and used for a legal identity authentication. Obfuscation techniques for location privacy were first presented by Duckham and Kulik in 2005. The idea of obfuscation is to degrade the information of a person’s location in order to obtain privacy [59]. Ref. [60] described how to perform obfuscation to preserve location privacy in a tracking system in real time. They implemented the obfuscator to map the true location of the user to an obfuscated location in real time, using services provided by a data server called the Global Data Plane. Ref. [48] introduced a new traceability ecosystem for the supply chain, based on the presence of privacy-sensitive information and the fact that actors are not willing to share their data openly. To achieve unlinkability, they use so-called stealth addresses. A stealth address is a privacy-enhancing technology for protecting the privacy of receivers of cryptocurrencies.

#### 5.1.3. Strategy 3—SEPARATE

With an emphasis on centralized web-based services, this strategy is often disregarded. However, personal data should be processed in a distributed fashion wherever possible. By separating the processing and the storage of different sources of personal data belonging to a person, it is no longer so easy to generate a complete profile of that person. Data from different sources should also be stored in different databases and these databases should not be linked [36]. In the IoT domain, distributed data processing is often implemented in WSNs. Therefore, pre-processing of, e.g., sensor data are already performed in the nodes and only relevant results are stored in the database. Distributed data processing also avoids large-scale centralized gathering and deters unauthorized access. The distributed storage of personal data limits the risk of privacy violation due malicious attacks and unauthorized access on the one hand, and privacy risks due to unconsented secondary knowledge discovery on the other hand [3]. One aspect of that strategy is covered in the principle of Use, Retention and Disclosure Limitation in ISO 29100. When PII is transferred internationally, PII controller should be cognizant of any additional national or local requirements [54]. Celosky et al. redefined that strategy as preventing correlation as much as possible by distributing or isolating any storage, collection or operation on personal data. The objective is to make sure that it is not possible to put together enough information about a data subject to endanger his or her privacy [55].

To implement the strategy SEPERATE, the fog computing paradigma can be used. Fog Computing extends the cloud computing paradigm to the edge of the network, thus making applications such as ATTS possible. Defining characteristics are: (a) low latency and location awareness, (b) Widespread geographical distribution, (c) mobility, (d) a very large number of nodes, (e) predominant role of wireless access, (f) a strong presence of streaming and real time applications, (g) heterogeneity [61]. In other words, fog computing is an additional layer to interact with the cloud. As with RTLS, many nodes communicate with the cloud eother through a server or directly. Furthermore, the privacy of users can be increased if data are already preprocessed on the fog level [8].

With the aim of preserving the privacy of the worker, the information about the position of the worker is exclusively locally managed and processed by the worker’s tablet. The ATTS in [50] provides a system of data and alarm sharing only in the case of emergency. Hepp et al. argue that RFID tags can be cloned, so it is not secure to use this technology in the supply chain. To solve this issue, they use the blockchain technology. However, one of the limitations of that technology is still the scalability of blockchain with regard to the number of transactions that can be processed by the network. Instead of individually logging each event on the blockchain, they use an additional blockchain-based timestamp API to maintain data integrity on the blockchain. This API called OriginStamp (https://originstamp.com/ (accessed on 20 February 2021)) aggregates events, combines them through hashing, and embeds this hash into a Bitcoin transaction. Once the network breaks the transaction into a block and validates it, the timestamp is considered unalterable proof of existence. With this approach, the integrity of the tracking data cannot be challenged [46].

#### 5.1.4. Strategy 4—AGGREGATE

This strategy states that personal data should be processed at the highest level of aggregation and with the least possible detail in which it is usable. The aggregation of personal data into groups with attributes restricts the level of detail in the remaining data. This has the effect that the data are less sensitive in relation to privacy [36]. Perera et al. defined the following guidelines that are relevant for that strategy: (A) To minimize knowledge discovery means to only discover personal data that are necessary to meet the primary objectives. (B) Geography-based aggregation is mainly used for location-based services. (C) Chain aggregation means that care is taken to ensure that data are no longer required for further processing and are not transferred to further nodes. (D) Time period-based aggregation - to reduce the granularity of data, they should be aggregated over time (e.g., days, weeks). (E) Category-based aggregation is helpful to further reduce granularity. Ref. [3]. The strategy AGGREGATE comprises the variations of “grouping” and “summarizing” at the coarsest granularity of data still useful for processing [55].

Ref. [42] used an ATTS to gather mobility data from people and vehicles. To ensure privacy, they aggregated the data in different categories, e.g., average speeds at different times of the day, at a specific node to forecast traffic jams.

Ref. [44] argues that crowd-sensing deployments, which feature rather long-term collection and storage of huge datasets, can become particularly attractive targets, which aggravates privacy risks. Their solution is inspired by the concept of mixed zones [62], in which quiet zones are introduced where users do not report their location. For outside observers, it is not possible to generate a motion profile.

### 5.2. Process-Oriented Strategies

#### 5.2.1. Strategy 5—INFORM

As the first process-oriented strategy, INFORM is related to the term of transparency. Whenever personal data are processed, the data subjects should be informed adequately. Furthermore, users should be informed about their data access rights and about how they can exercise them [36]. This information step can be taken at any stage of the data life cycle and can be grouped in two categories: pre-inform and post-inform. The pre-inform step occurs before personal data are entered, while the post-inform step takes place after data leave a specific life cycle phase [3]. The ISO 29100 standard refers to the principles “Consent and Choice” and “Purpose Legitimacy and Specification” in the strategy INFORM. They state that the users should always have a choice about whether or not to allow the processing of their PII. The choice for opt-in must be given freely, specific and on a knowledgeable basis [54].

When it comes to tracking systems, this means that wherever people are being tracked, they should be informed about which data are collected, processed and disseminated. The tracked areas should be marked with warning signs if these areas are public. In this way, every present individual can decide freely whether he or she consents to being tracked.

In [37], the visitors of the tracked building are equipped with a visitor pass that implements an RFID tag. They do not state whether the visitors are informed about the fact that they are tracked. In that case, it is a serious offence against the privacy strategy INFORM.

#### 5.2.2. Strategy 6—CONTROL

This strategy states that data subjects should be informed about the processing and storing of their personal data. Transparent information about the security and access to design documentation is also a good practice. Furthermore, data subjects should be informed about the use of their data by third parties and about the type of information they receive. Additionally, it is important to inform data subjects about their data access rights and how to execute them [36]. A control mechanism should allow data owners to manage their privacy configuration in an simple and time-saving way [3]. It should also be mentioned that Cavoukian’s second foundational principle “Privacy as default setting”, the author states that whenever privacy settings can be made, the least invasive setting should always be used as the default setting [24]. In addition to the suggestions from [3,36], it has to be considered that the data controller should provide choices for the purposes of limiting the processing, accessing, correcting and removing their information [54]. Celosky et al. also refer to the right of self-determination [63], and to keep personal information up to date and accurate. What distinguishes CONTROL and INFORM from other policies is that they focus on the data subject and the controller.

Transparent information about the security to design documentation is good practice with reference to the CONTROL strategy. Refs. [10,12,37,41,45,47,48,49] provide detailed information about their security analysis and thread models. Using these systems, it is clear which privacy issues have been taken into account, so these systems can be better adapted to individual conditions. In [39] an ATTS yields location data from the staff of a university. To refer to the strategy CONTROL, they made it possible for the staff to overrule the system and set the location by themselves in a desktop application. In this way, the user has full control over the system’s output.

#### 5.2.3. Strategy 7—ENFORCE

This strategy states that a privacy policy compatible with legal requirements should be in place and should be enforced. It assures that a privacy policy is in place within the legal requirements [36]. More specifically, it means ensuring the most comprehensive commitment to creating, maintaining, and upholding policies and technical controls regarding the storage, collection, disclosure, modification, and breaches of personal data within the limits of the agreed purposes. In this context, the following tactics should be considered:

- Create—to respect the value of privacy and decide upon policies which share that value [64].
- Maintain—to respect the policies when designing or modifying features, especially after updating the policies to better protect personal information [65].
- Uphold—to ensure compliance with these policies. Personal information is valued as an asset and privacy as a goal to incentivize as a critical feature [66].

The articles found for this review are all scientific papers, therefore no information on the planned handling during continuous operation is given. However, ref. [7] for example refers to the consideration of existing laws such as the GDPR, while others such as [10,38,42] refer to the local legislation of the country where the system is to be applied.

#### 5.2.4. Strategy 8—DEMONSTRATE

The last strategy DEMONSTRATE requires the data controller to comply with the privacy policy and any applicable legal requirements and to be able to demonstrate this to data subjects. This strategy goes one step further than ENFORCE, in so far as the responsible person must be able to prove control of the system. In the case of complaints or problems, the data controller should be able to determine the extent of any possible privacy breaches [36]. This strategy was defined more precisely within the framework of Perera et al. They divided the DEMONSTRATE strategy into eight practical guidelines [3]

- Information disclosure refers to the fact that the data controller should be informed exactly about the acquisition, collection, storage and processing of privacy data.
- A privacy-preserving ATTS should log all events where personal information is gathered, stored, processed or disseminated.
- A systematic and independent examination of logs, procedures, processes, software and hardware specifications should be performed.
- A form of compliance demonstration is Open Source.
- Data flow diagrams make it possible for interested parties to see the flows of data within an IoT application.
- Certification by a neutral institution increases the trustworthiness of IoT applications.
- To demonstrate privacy protection, a good practice is to use industry-wide standards that inherit privacy protection capabilities.
- Depending on the country and region, different guidelines, laws and regulations must be observed for IoT applications.

ISO 29100 deals with this strategy in its privacy principles including the following points:

- Openness, transparency and notice—this principle means to disclose the choices offered by the data controller to the data subjects for the purposes for accessing, correcting and removing their information.
- Information security—access to personal data shall be granted only to those who need it in order to perform their duties.
- Privacy compliance—applicable laws can provide that supervisory authorities are responsible for monitoring compliance with applicable data protection laws [54].

Celsoky et al. define the strategy DEMONSTRATE as ensuring as much evidence as possible for testing, auditing, logging, and reporting on policies and technical controls regarding storage, collection, retention, sharing, changes, breaches or operation on personal data, in a timely manner, within the constraints of the agreed-upon purposes. Furthermore, they provide the following tactics: (A) Log, (B) Audit, and (C) Report.

In terms of employee privacy, it is important to know when the system was in operation and who made changes to the configuration or the stored data. Otherwise, it could be possible to manipulate data records in such a way that it could result in disadvantages for employees, or unauthorized interpretations could be carried out. To avoid this, ref. [7] implemented a logging system that stores all changes of the configuration and the database. This makes it possible to check afterwards who queried which data to avoid privacy issues. To restrict unauthorized access to private information while providing healthcare services, ref. [43] introduced privacy-preserving access control. They use a role-based access control mechanism in combination with RFID to restrict unauthorized access to private data. Almost every article we examined provided an exact description of the data flow, as can be seen in Table 6. This is, of course, due to the fact that these are scientific papers and therefore these aspects are examined in detail.

## 6. Discussion

To answer the research questions, a literature review was conducted using the approach described in Section 3. The articles found were evaluated using Perera’s PdB Guidelines. In the following chapter, each research question is answered separately.

### 6.1. Research Questions

What are the most common reasons to implement privacy features in ATTS?

**Figure 3:**
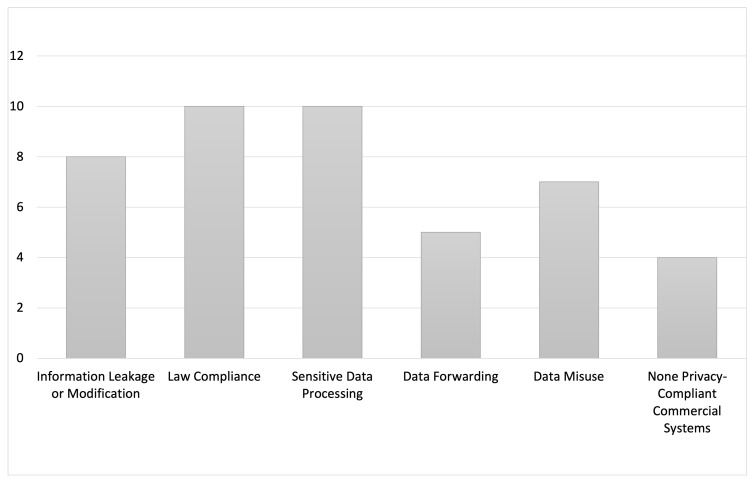
Reasons why privacy is implemented in ATTS in terms of frequency of occurrence.

To answer those research questions, we collected all arguments from the authors as to why privacy features were implemented in those ATTS and generated the following categories: (1) *Information Leakage or Modification* (2) *Law Compliance* (3) *Sensitive Data Processing* (4) *Data Forwarding* (5) *Data Misuse* (6) *Non privacy-Compliant Commercial Systems*. In Table 4 a overview between the articles an the respective reasons for privacy features can be found. None of the articles gave an argument on every category. The authors mostly gave three or four reasons why privacy was an important part of the development phase. Figure 3 shows an overview how often which reason was mentioned in total. “Law Compliance”, “Information Leakage or Modification” and “Sensitive Data Processing” were the most stated reasons. Due to data protection regulations (e.g., GDPR in the EU) this is becoming more and more important and system developers, who process personal data are urged to adhere to these regulation. Perera [67] has shown that by demonstrating privacy strategies, guidelines and patterns, developers become more aware and systems become more privacy friendly. The results of this work also show that this awareness exists in the scientific community but maybe not so significantly in the industry.

Which approaches exist to evaluate privacy of users in ATTS?

To evaluate the privacy of the chosen systems, it was decided to use the framework of Perera [3]. It is based on already established guidelines such as Hopeman [36] and Cavoukian [24], but allowed a more detailed analysis of the data-related and process-related privacy considerations of an IoT system. With the guidlines of Perera et al. [3], ATTS can be specifically described in terms of privacy. This framework divides the data life cycle into five phases: Consent and Data Acquisition, Data Pre-Processing, Data Processing and Analysis, Data Storage, and Data Dissemination. Since the architecture of an ATTS usually corresponds to that of a typical IoT system, the data-oriented guidelines give a clear overview of which features in terms of privacy have been implemented in the different lifecycle-phases and which privacy features are not implemented. On the other hand, the process-oriented guidelines are only suitable for evaluation to a limited extent, since they depend strongly on the use case of the analysed ATTS. In particular, when applied in the working environment, it is usually not possible for users to proactively decide for or against the collection of data by the employer. To meet these requirements, it would be necessary to include the transparency of the organization as a factor in the evaluation.

To determine the level of privacy in a system, a privacy risk analysis is necessary in advance to define the expected privacy issues [68]. For ATTS, it is particularly important whether people, objects or both are tracked by the system, since the importance of privacy changes accordingly. If people are being tracked, then the highest privacy standards must be adhered to; for asset tracking systems, the process-oriented guidelines can be compromised. However, it must be ensured that this data collection does not lead to people being tracked unknowingly (e.g., by smartphones or fitness tapes). In addition, it must be ensured that the data are not aggregated in a way that makes it possible to monitor individuals (e.g., metadata analysis).

What are the most frequently used strategies implementing privacy features in ATTS?

**Figure 4:**
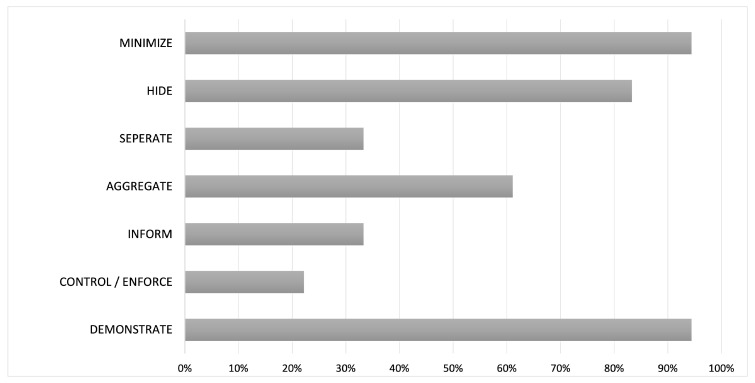
Strategies from [36], which were implemented most frequently in the analyzed articles (in percent).

According to Hoepman, the data-oriented strategy MINIMIZE is the most important and the easiest to implement [36]. Nearly every analyzed article took this strategy into consideration in the design phase. In each case, only the data necessary to achieve the specified task were collected, stored and processed. Basically, it seems that data-oriented guidelines were implemented more often than process-oriented ones, as shown in Figure 4. The DEMONSTRATE strategy, however, was also used in almost all systems. Important guidelines for ATTS in relation to the DEMONSTRATE strategy are logging, auditing and legal compliance. Logging is intended to prevent data from being unlawfully altered or passed on. Therefore, according to the result that law compliance is one of the major reasons to implement privacy features into ATTS, this strategy is related to that. The HIDE strategy was also often implemented in the found articles. Encryption and anonymization are actually standard in most transmission technologies and can be implemented with little effort.

Depending on the system architecture and the further use of the data, the SEPARATE strategy will be applied to the investigated ATTS. However, it is important to strictly separate the recorded data from particularly sensitive personal data such as the healthcare data of data subjects. An interesting possibility is to link the determined tracking data with the blockchain. The advantages of this would be transparency, trust and unchangeability. Important methods to protect the user privacy of ATTS are offered by the strategy AGGREGATE. ATTS in particular generate large and high-resolution data volumes in a very short time. For this purpose, it is important to aggregate the data as early as possible in categories or time periods.

The INFORM and CONTROL strategies have similar approaches related to ATTS and are probably the two most important process-oriented strategies. To achieve a high acceptance of ATTS in the working environment, it is important that employees are informed transparently about the benefits of the systems. Furthermore, it should be possible for employees to deactivate tracking in certain cases. The strategy ENFORCE is to be understood as a constant process in the operation of IoT systems, and especially the evaluation of privacy should be carried out anew when the objectives of the systems change. Especially with regard to tracking systems, which primarily serve the purpose of traceability, a multitude of possible evaluations of the data are conceivable. Important guidelines for ATTS in relation to the DEMONSTRATE strategy are logging, auditing and legal compliance. Logging is intended to prevent data from being unlawfully altered or passed on.

### 6.2. Limitations

The findings of this study have to be seen in light of some limitations. The first is the fact that privacy is an abstract concept and must always be considered in the interplay between the regionally applicable laws. In particular, in the area of tracking systems in the work environment, the legal requirements are not fully clarified. For example the General Data Protection Regulation (GDPR) is a regulation enacted by the European Parliament and Council which primarily aims to give control back to citizens and residents over their personal data. However, the impact of the GDPR on employee tracking must be decided depending on the application. Companies are well advised to act privacy friendly in order to avoid penalties. In this review, no commercial systems were used, only ATTS from scientific projects, which were specifically tailored to the intended use case and therefore privacy features were implemented in a very selective way. In the case of commercial systems, it can be assumed that these systems are usually developed for a broad range of applications and therefore they have fewer privacy features implemented. Our aim here was not to show a particularly large number of privacy weaknesses, rather to present various options for protecting privacy in ATTS. Another limitation is that the chosen articles mostly reference to the data-oriented guidelines. The process-oriented guidelines were only mentioned in passing or omitted completely in most analyzed articles. Another limiting factor is the number of found articles. We were searching for ATTS which implemented privacy features and had a strong emphasis on this in scientific articles. A lot of the papers dealt with the topic in a wider context such as general guidelines, specific privacy issues without ATTS or privacy preserving protocols. Furthermore, many papers on tracking systems were found in which the search terms were present but the topic of privacy was not discussed in detail.

### 6.3. Future Perspectives

In this survey, the reasons why privacy is used in ATTS and which technologies and methods are used to protect the privacy of the users, especially in the work environment, were determined. Based on this survey, guidelines and patterns can be designed to protect the privacy of users in ATTS. Furthermore, it was shown that privacy guidelines for IoT applications are also suitable for ATTS, but should be adapted slightly, especially the process-oriented strategies must rely strongly to the application. Another important point to improve privacy in ATTS is to develop methods to evaluate ATTS multicriterially and quantitatively with respect to privacy. This is important because each application of ATTS has different privacy requirements. For example, in asst tracking systems it is important to note that employee data e.g., data from smartphones or fitness wristbands are not recorded. Whereas in systems for increasing occupational safety, it is necessary to collect employee data, but, according to the MINIMISE strategy, only as much is absolutely necessary for the task fulfillment. Furthermore, system developers should receive clear and precise information to implement privacy in ATTS. Perera has already shown that there is potential for increasing awareness to privacy in IoT systems [67].

Another important point that researchers should consider in the future is a clearer separation between security and privacy. While the concepts of security and privacy overlap, they are not the same. Data security ensures users that their data are not seen by anyone with unauthorized access. It should be distinguished from data privacy, which is an active method of controlling the access to personally identifiable information. Data that is properly secured through encryption can still reveal a user’s identity by being shared or sold to third parties [69].

## 7. Conclusions

This survey presents a systematic literature review about privacy features in ATTS. In a first step we examined the motives why deleoper implemented privacy features in their systems. We categorized the different statements into six categories: (1) Information Leakage and Modification, (2) Law Compliance, (3) Sensitive Data Processing, (4) Data Forwarding, (5) Data Misuse, and (6) Non privacy-Compliant Commercial System.

The most commonly used argumentation was the need to comply with the law (Law Compliance). As tracking systems can store and process personal data of employees, it is also necessary to comply with local laws and regulations. Otherwise, as for example in Europe through the GPDR, the operators of these systems can risk high financial penalties. Developers are increasingly required to react to this situation to make their systems secure in terms of privacy. This is also the case in the category ‘Non privacy-Compliant Commercial Systems’, where tracking systems had to be developed on their own because the systems on the market did not meet the own privacy requirements. In many cases, ATTS process sensitive data of employees, e.g., they enable electronic monitoring of the performance and behavior of employees. This could lead to a subjective feeling of supervision among employees and may create increased uncertainty. The process-oriented strategies are particularly suitable for dealing with these uncertainties on the part of employees, as they are intended to specifically promote the transparency of systems. We used the guidelines of Perera et al. [3] to evaluate ATTS which were developed in scientific projects that had a strong reference to privacy. It turned out that it is generally possible to use the guidelines that were developed for IoT systems also for ATTS. In particular, the data-oriented policies proved to be very helpful. However, the process-oriented guidelines are very general and rather tailored for applications with unknown users. In ATTS, however, there is often a relationship between the operator and the user of the system, which has an impact on the trust between the parties. Therefore, it would be important to go into more detail about the role of the “employe”. We have shown that the data-oriented strategies MINIMISE and HIDE are most frequently used in the evaluated systems. Furthermore, the strategy DEMONSTRATION was also used very often. This strategy includes the policy compliance. This fits well with the results from RQ1, where a frequently cited reason for privacy features is legal certainty. In terms of future work, we can consider a privacy evaluation framework especially for ATTS. This framework could specifically implement the important requirements and technical details of tracking systems. This would simplify the evaluation of these systems, and therefore more technicians would develop privacy-preserving ATTS. Also, user roles should be adaptable in these guidelines. Easier evaluations and privacy preserving ATTS systems could lead to more end user privacy without compromising the added value of these systems.

## Figures and Tables

**Table 1:** Overview of common IPS technologies according to Zafari [13] (valid for all entries without additional reference).

Radio Standard	Range Indoor	Power Consumption	Location Accuracy	Privacy Risks	Advantage	Disadvantage
Wifi	35 m	moderate	m [14]	Smartphones or fitness wristbands with activated Wifi could also be tracked [15,16]	Widely available, high accuracy, no extra hardware	Prone to noise, requires complex process algorithms
UWB	10–20 m	moderate	cm–m [14]	Low risk because of seperated hardware	Iimmun to interference, very high accuracy	Shorter range, requires specific hardware, high costs
RFID	200 m	low	dm–m [14]	Smartcards of employees could be tracked [15,17]	Iow power, different possibilities for range (active, passive)	Localization accuracy is low
Bluetooth	100 m	low	5–10 m [18]	Smartphones or fitness wristbands with activated Bluetooth could also be tracked [15]	High throughput, reception range, low energy consumption	Low localization accuracy, prone to noise
LoRA	500 m	extremely low	10–20 m [19]	Low risk because of seperated hardware	Wide reception range, low energy consumption	Long distance between base station and device, server outdoor-to-indoor signal attenuation due building walls

**Table 2:** Overview from inclusion and exclusion criteria for this review.

Inclusion Criteria	Exclusion Criteria
articles that present asset tracking or employee tracking systems with strong emphasis to privacy. the described system implements privacy features or inherits the Privacy by Design concept as well as there are also privacy issues are explained the system is designed for applications in industry, health care, agricultural environments or smart cities.	tracking of digital assets design concepts for tracking systems, but no developed system articles about special privacy features in systems articles related to indoor navigation systems articles, where the subject (e.g., assistance systems in manufacturing) is described as possible future solutions, related work or in a very broad context

**Table 3:** Articles which were used for this review.

Author	Year	Publication Type	Conference/Jounal	Publisher	Technology
Buccafurri et al.	2014	Conference	Int. Computer Software and Applications Conf.	IEEE	RFID
Baslyman et al.	2015	Journal	Personal and Ubiquitous Computing	Springer Link	Wifi
Stone and Spies	2015	Conference	Int. Conf. on Computing, Communication and Security (ICCCS)	IEEE	Presence Sensors
Kim et al.	2016	Conference	Int. Parallel and Distributed Processing Symp.	IEEE	Wifi
Martin et al.	2016	Conference	Int. Conf. on Bioinformatics, Computational Biology, and Health Informatics	ACM	Bluetooth
Fernandez-Ares et al.	2016	Journal	Future Generation Computer Systems	Scinece Direct	Wifi, Bluetooth
Moniem et al.	2017	Conference	Ubiquitous, Autonomic and Trusted Computing, UIC-ATC	IEE	RFID
Rahman et al.	2017	Journal	Future Generation Computer Systems	Science Direct	RFID
Ziegeldorf et al.	2017	Conference	Conf. on Wireless On-demand Network Systems and Services (WONS)	IEEE	Wifi/Bluetooth
Ashur et al.	2018	Conference	Int. Conf. on Cryptology and Network Security	Springer Link	LoRa, Bluetooth
Hepp et al.	2018	Conference	Workshop on Cryptocurrencies and Blockchains for Distributed Systems	ACM	Blockchain, RFID
Pešić et al.	2018	Conference	Int. Conf. on Web Intelligence, Mining and Semantics	ACM	Bluetooth
Salman et al.	2018	Conference	Glob. Conf. on Internet of Things (GCIoT)	IEEE	Wifi
Anandhi et al.	2019	Journal	Wireless Personal Communications	Springer Link	RFID
Jandl et al.	2019	Conference	Int. Conf. on emerging tech. and factory automation	IEEE	Bluetooth
Maouchi et al.	2019	Conference	Symp. on Applied Computing	ACM	Blockchain
Buccafurri et al.	2020	Journal	Trans. on Services Computing	IEE	RFID
Faramondi et al.	2020	Conference	Int. Convention on Information, Communication and Electronic Technology	IEEE	Wifi, Bluetooth

**Table 4:** Reasons why privacy features or the PbD concept were implemented (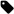: = Objects, 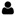: = Persons, 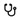: = Healthcare, 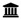: = University, 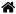: = Smart Home, 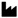: = Industry, 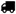: = Logistics).

	[37]	[38]	[39]	[40]	[41]	[42]	[12]	[43]	[44]	[45]	[46]	[8]	[10]	[47]	[7]	[48]	[49]	[50]
Tracked Items	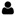	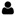	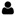	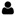	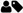	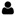	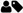	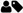	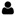	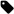	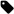	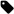	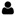	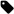	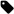	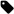	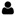	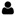
Tracking Technology	RFID	WiFi	-	WiFi	BT	WiFi/BT	RFID	RFID	WiFi/BT	BT	Blockchain	BT	WiFi	RFID	BT	RFID	RFID	WiFi/BT
Application Domain	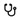	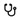	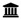	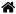	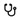	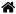	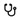	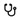	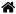	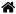	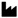	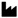	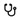	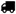	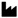	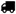	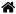	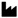
**Reasons for Privacy Features**																		
Leakage or Modification	-	**✓**	-	-	**✓**	-	**✓**	**✓**	-	**✓**	**✓**	**✓**	-	**✓**	-	-	-	-
Law Compliance	**✓**	**✓**	**✓**	-	-	**✓**	-	-	**✓**	-	-	-	**✓**	-	**✓**	**✓**	**✓**	**✓**
Sensible Data Processing	-	-	-	-	**✓**	**✓**	**✓**	**✓**	**✓**	**✓**	-	**✓**	-	-	**✓**	-	-	**✓**
Data Forwarding	-	-	-	-	-	-	-	**✓**	**✓**	-	**✓**	**✓**	-	-	-	**✓**	-	-
Data Misuse	-	-	**✓**	-	**✓**	-	-	**✓**	**✓**	-	**✓**	-	-	-	**✓**	-	-	**✓**
None-Privacy Comm. System	**✓**	**✓**	-	-	-	-	-	-	-	-	-	-	-	-	**✓**	-	**✓**	-

**Table 5:** Guidelines by Perera et al. [3] for data-oriented strategies applied to the selected articles.

	[37]	[38]	[39]	[40]	[41]	[42]	[12]	[43]	[44]	[45]	[46]	[8]	[10]	[47]	[7]	[48]	[49]	[50]
MINIMIZE																		
Minimise Data Acquisition	**✓✓**	**✓✓**	**✓✓**	**✓✓**	**✓**	**✓✓**	**✓✓**	**✓✓**	**✓✓**	**✓**	**✓✓**	-	**✓✓**	-	**✓✓**	**✓**	**✓✓**	**✓✓**
Minimise Number of Data Sources	**✓**	**✓**	**✓✓**	**✓✓**	**✓✓**	**✓✓**	**✓**	**✓✓**	**✓✓**	**✓✓**	**✓✓**	**✗**	**✓**	**✓**	**✓✓**	-	**✓✓**	**✗**
Minimise Raw Data Intake	**✓✓**	**✓✓**	**✓✓**	**✓✓**	**✓✓**	**✓✓**	-	**✓✓**	-	**✓✓**	**✓✓**	-	**✓✓**	-	**✓✓**	-	**✓✓**	**✓✓**
Minimize Knowledge Discovery	**✓✓**	**✓✓**	**✓✓**	**✓✓**	**✓✓**	**✓✓**	**✓✓**	**✓✓**	**✓✓**	**✓✓**	**✓✓**	**✗**	**✓✓**	**✓✓**	**✓✓**	-	**✓✓**	**✓✓**
Minimize Data Storage	-	**✓✓**	**✓✓**	**✓✓**	**✓✓**	**✓**	**✓✓**	-	-	**✓✓**	**✓✓**	**✓✓**	**✓**	**✗**	**✓**	-	-	**✓✓**
Minimize Data Retention Period	-	**✓✓**	**✓**	-	**✗**	**✗**	**✓✓**	**✗**	**✗**	**✓**	**✗**	-	**✓✓**	**✗**	**✓**	**✗**	-	**✓✓**
Query Answering	-	**✗**	**✓✓**	**✓✓**	**✓✓**	**✓**	-	**✓✓**	**✓✓**	-	-	-	-	-	-	-	**✓✓**	**✓✓**
Repeated Query Blocking	-	**✗**	**✗**	**✗**	**✓**	-	-	-	-	**✓✓**	-	-	-	-	-	-	-	-
Minimize Data Retention Period	-	**✓✓**	**✓**	-	**✗**	**✗**	**✓✓**	**✗**	**✗**	**✓**	**✗**	-	**✓✓**	**✗**	**✓**	**✗**	-	**✓✓**
HIDE																		
Hidden Data Routing	**✓**	**✓**	**✗**	**✗**	**✗**	**✗**	**✗**	**✓✓**	**✓✓**	**✓**	-	**✗**	**✗**	**✗**	**✗**	**✓✓**	**✓✓**	-
Data Anonymization	**✓✓**	**✓**	**✗**	**✗**	**✓**	**✓✓**	**✓✓**	**✓✓**	**✓✓**	**✓✓**	**✓✓**	**✗**	**✓✓**	**✗**	**✓✓**	**✓✓**	**✓✓**	**✓✓**
Encrypted Data Communication	**✓✓**	**✗**	**✓✓**	**✓✓**	**✓✓**	**✓✓**	**✓✓**	**✓✓**	**✓✓**	**✓✓**	**✓**	**✗**	**✓✓**	**✗**	**✗**	**✓✓**	**✓✓**	**✓✓**
Encrypted Data Processing	**✓✓**	**✗**	**✗**	**✗**	**✓✓**	**✓**	**✓✓**	**✓✓**	**✓✓**	**✓✓**	**✓✓**	**✗**	**✓✓**	**✗**	-	**✓✓**	**✓✓**	**✗**
Encrypted Data Storage	-	**✗**	**✗**	**✗**	**✓✓**	**✓✓**	**✗**	-	-	**✓✓**	**✓✓**	**✗**	**✓✓**	**✗**	-	**✓✓**	-	**✓✓**
SEPERATE																		
Distributed Data Processing	**✓**	**✓**	**✗**	**✗**	**✗**	**✓✓**	**✗**	-	**✗**	**✗**	**✓✓**	**✓✓**	**✗**	**✗**	**✓✓**	**✓✓**	-	**✓✓**
Distributed Data Storage	-	**✓**	**✗**	**✗**	**✓✓**	**✓✓**	**✗**	-	**✗**	**✗**	**✓✓**	**✓✓**	**✗**	**✗**	**✗**	**✓✓**	-	**✓✓**
AGGREGATE																		
Knowledge Discovery Based Aggregation	-	**✗**	**✗**	**✗**	**✓✓**	**✓✓**	-	**✓✓**	**✓✓**	-	-	**✗**	-	-	**✓✓**	**✓✓**	-	-
Geography Based Aggregation	-	**✗**	-	-	**✓✓**	**✓✓**	**✗**	**✓✓**	**✓✓**	**✓✓**	-	**✓**	-	-	**✓✓**	**✗**	-	**✓✓**
Chain Aggregation	-	-	-	-	**✓✓**	**✓✓**	**✗**	**✓✓**	**✓✓**	**✓✓**	**✓✓**	**✓✓**	-	**✗**	**✓✓**	-	-	-
Time Period Based Aggregation	-	**✗**	-	-	**✗**	**✓**	**✓**	**✓✓**	**✓**	**✗**	**✗**	**✓**	**✗**	**✗**	**✓✓**	-	-	-
Category Based Aggregation	-	**✗**	-	-	**✓**	**✓**	**✓✓**	**✓✓**	**✓✓**	**✗**	-	**✗**	**✓**	-	**✓✓**	-	-	-

**Table 6:** Guidelines by Perera et al. [3] for process-oriented strategies applied to the selected articles. (* The guideline for the CONTROL and ENFORCE strategies [3] in the framework are identical in their meaning, which is why we only list one).

		[37]	[38]	[39]	[40]	[41]	[42]	[12]	[43]	[44]	[45]	[46]	[8]	[10]	[47]	[7]	[48]	[49]	[50]
INFORM	Information Disclosure	**✗**	**✓**	**✓**	**✓**	**✓✓**	**✗**	-	-	**✓**	-	-	**✓**	**✓**	-	-	-	**✓**	**✓✓**
CONTROL/ENFORCE *	Control	**✓**	**✓**	**✓**	**✗**	**✓**	**✗**	**✓**	**✓**	**✓**	**✓**	**✗**	**✗**	**✓**	**✓**	**✓**	**✓**	**✓**	**✓✓**
DEMONSTRATE	Logging	**✓**	**✓✓**	**✗**	**✗**	**✓✓**	**✗**	**✗**	**✗**	**✗**	**✗**	**✓✓**	**✗**	-	**✗**	**✓✓**	**✓✓**	-	**✓✓**
	Auditing	-	-	**✓✓**	**✗**	-	**✗**	**✗**	**✗**	**✗**	**✗**	-	**✗**	-	**✗**	-	-	-	-
	Opensource	**✓**	**✗**	**✓**	**✓**	**✗**	**✗**	**✗**	**✗**	**✗**	**✗**	**✗**	**✗**	**✗**	**✗**	**✗**	**✗**	**✗**	**✗**
	Data Flow Diagrams	**✗**	**✓✓**	**✓✓**	**✓✓**	**✓✓**	**✓✓**	**✓✓**	**✓✓**	**✓✓**	**✓✓**	**✓✓**	**✓✓**	**✓✓**	**✓✓**	**✗**	**✗**	**✓✓**	**✓✓**
	Certification	-	**✗**	**✗**	**✗**	**✓**	-	**✗**	-	-	**✗**	-	**✓✓**	**✗**	**✗**	**✗**	**✓✓**	-	**✗**
	Standardization.	-	**✗**	**✗**	**✗**	-	**✓**	**✗**	-	**✓**	**✗**	**✓**	**✓**	**✗**	**✗**	-	**✗**	-	-
	Compiliance	-	**✗**	**✗**	**✗**	**✓**	**✓✓**	**✗**	**✓✓**	**✓✓**	**✗**	-	**✗**	**✗**	**✗**	**✓✓**	**✗**	**✓✓**	**✓✓**

## Data Availability

Data supporting reported results can be found on GitHub (https://github.com/CJ1979/sensitrack).
